# Sphingosine 1-Phosphate-Upregulated COX-2/PGE_2_ System Contributes to Human Cardiac Fibroblast Apoptosis: Involvement of MMP-9-Dependent Transactivation of EGFR Cascade

**DOI:** 10.1155/2022/7664290

**Published:** 2022-02-22

**Authors:** Chien-Chung Yang, Li-Der Hsiao, Ya-Fang Shih, Mei-Hsiu Su, Chuen-Mao Yang

**Affiliations:** ^1^Department of Traditional Chinese Medicine, Chang Gung Memorial Hospital at Tao-Yuan, Kwei-San, Tao-Yuan 33302, Taiwan; ^2^School of Traditional Chinese Medicine, College of Medicine, Chang Gung University, Kwei-San, Tao-Yuan 33302, Taiwan; ^3^Department of Pharmacology, College of Medicine, China Medical University, Taichung 40402, Taiwan; ^4^Ph.D. Program for Biotech Pharmaceutical Industry, China Medical University, Taichung 40402, Taiwan; ^5^Department of Post-Baccalaureate Veterinary Medicine, College of Medical and Health Science, Asia University, Wufeng, Taichung 41354, Taiwan

## Abstract

Human cardiac fibroblasts (HCFs) play key roles in normal physiological functions and pathological processes in the heart. Our recent study has found that, in HCFs, sphingosine 1-phosphate (S1P) can upregulate the expression of cyclooxygenase-2 (COX-2) leading to prostaglandin E_2_ (PGE_2_) generation mediated by S1P receptors/PKC*α*/MAPKs cascade-dependent activation of NF-*κ*B. Alternatively, G protein-coupled receptor- (GPCR-) mediated transactivation of receptor tyrosine kinases (RTKs) has been proved to induce inflammatory responses. However, whether GPCR-mediated transactivation of RTKs participated in the COX-2/PGE_2_ system induced by S1P is still unclear in HCFs. We hypothesize that GPCR-mediated transactivation of RTKs-dependent signaling cascade is involved in S1P-induced responses. This study is aimed at exploring the comprehensive mechanisms of S1P-promoted COX-2/PGE_2_ expression and apoptotic effects on HCFs. Here, we used pharmacological inhibitors and transfection with siRNA to evaluate whether matrix metalloprotease (MMP)2/9, heparin-binding- (HB-) epidermal growth factor (EGF), EGF receptor (EGFR), PI3K/Akt, MAPKs, and transcription factor AP-1 participated in the S1P-induced COX-2/PGE_2_ system determined by Western blotting, real-time polymerase chain reaction (RT-PCR), chromatin immunoprecipitation (ChIP), and promoter-reporter assays in HCFs. Our results showed that S1PR1/3 activated by S1P coupled to G_q_- and G_i_-mediated MMP9 activity to stimulate EGFR/PI3K/Akt/MAPKs/AP-1-dependent activity of transcription to upregulate COX-2 accompanied with PGE_2_ production, leading to stimulation of caspase-3 activity and apoptosis. Moreover, S1P-enhanced c-Jun bound to COX-2 promoters on its corresponding binding sites, which was attenuated by these inhibitors of protein kinases, determined by a ChIP assay. These results concluded that transactivation of MMP9/EGFR-mediated PI3K/Akt/MAPKs-dependent AP-1 activity was involved in the upregulation of the COX-2/PGE_2_ system induced by S1P, in turn leading to apoptosis in HCFs.

## 1. Introduction

Cardiac fibroblasts, one kind of the main cell types in the cardiac tissue, play key roles in normal myocardial function and myocardial remodeling, including myofibroblast differentiation, proliferation, migration, secretion of cytokines and growth factors, matrix protein synthesis, and inflammation [1]. Cyclooxygenase- (COX-) 2, one of two kinds of COXs, could be inducible during various inflammatory conditions by several proinflammatory factors leading to prostaglandin (PG) synthesis in various types of cells. Although the precise role of COX-2 in cardiac physiology and diseases remains not fully understood and controversial, there are progressively increased reports showing that pharmacological inhibitors of COX-2 could reduce recurrent angina, myocardial infarction, and death [2]. Hence, COX-2 might have a pivotal role in inflammatory heart diseases, which could be an important anti-inflammatory target [3]. Sphingosine 1-phosphate (S1P), one of the bioactive metabolized products of sphingolipids, can modulate a lot of functions of physiology and pathology, including proliferation, inflammation, and apoptosis [4–6]. Moreover, the elevated levels of S1P are associated with heart failure [7] and postmyocardial infarction and cardiac inflammation [8]. Moreover, S1P/S1P1 receptor cascade may be involved in angiogenesis and vasodilatation in the vasculature, activated by the estrogen/estrogen receptor leading to expression of nitric oxide synthase through activation of Akt in endothelial cells [9]. Previously, some studies showed that S1P could induce PGE_2_ synthesis related to the upregulation of COX-2 in numerous types of cells and organs [10–14]. We recently reported that S1P induces the expression of COX-2/PGE_2_ through NF-*κ*B activity enhanced by PKC*α*-mediated mitogen-activated protein kinases (MAPKs) in HCFs [15]. Therefore, S1P could exert a key role in cardiovascular inflammatory disorders.

S1P regulates the cellular functions mediated through S1P receptors 1-5, one kind of G-protein coupled receptor (GPCR), but only S1PR1-3 are expressed on the cardiovascular systems [16, 17]. Both S1PR1-coupled G_i_ and S1PR3-coupled G_q_ activate phospholipase C (PLC) and consequently induce Ca^2+^ mobilization and PKC activity [18]. Our recent study has found that S1P-induced responses are mediated through S1PR1/3 coupled by either G_i_ or G_q_ protein leading to PKC*α*-dependent MAPK activation in HCFs [15]. Several GPCRs have been recognized to transactivate receptor tyrosine kinases (RTKs) and non-RTKs including EGFR, PDGFR, c-Src, and Pyk2 mediated through the shedding of cell-surface proteins such as heparin-binding- (HB-) EGF by matrix metalloproteases (MMPs) [19]. The present study also investigated whether GPCR-mediated transactivation of RTKs is involved in the S1P-induced COX-2 expression and PGE_2_ production in HCFs.

The transactivation of EGFR modulated downstream protein kinases including phosphatidylinositol-3-kinase (PI3K)/Akt and MAPKs which could play critical roles in various cellular functions and pathogenesis, such as proliferation, migration, and inflammation. Further, S1PR1/3 have been shown to couple to G_i_ or G_q_ relaying the signaling through Ras/p42/p44 MAPK and PI3K/Akt. Numerous studies have implied that the S1P-induced COX-2/PGE_2_ expression is mediated through c-Src, EGFR, PI3K/Akt, MAPKs, and transcription factors in a variety of cells [10, 12]. Our earlier research indicated that S1P could stimulate MAPKs-dependent AP-1 activation leading to increased expression of COX-2 in human tracheal smooth muscle cells (HTSMCs) [12]. Therefore, both upstream mechanisms of MMP activation and downstream signaling components regulated by S1P-stimulated EGFR transactivation are further differentiated in HCFs. Further, activation of EGFR, PI3K/Akt, and MAPKs leading to AP-1 activity enhancing the expression of COX-2 induced by S1P was also evaluated.

Several proinflammatory factors stimulating upregulation of the inflammatory mediators play critical roles in heart diseases [20, 21]. However, in HCFs, the detailed mechanisms of COX-2 upregulation and release of PGE_2_ induced by S1P were not fully defined. Although S1P generally elicits mitogenic and antiapoptotic effects, some evidence shows that S1P has apoptotic and growth-inhibitory effects related to the caspase-3 pathway [22, 23]. Therefore, the present study also dissected the detailed mechanisms by which S1P promoted COX-2/PGE_2_ expression leading to apoptotic effects on HCFs. In the present study, these discoveries revealed that S1P-triggered upregulation of the COX-2/PGE_2_ axis is, at least partially, caused by S1PR1/3 coupled to G protein either G_i_ or G_q_, EGFR transactivation, and activation of PI3K/Akt-dependent signaling components of JNK1/2, p42/p44 MAPK, p38 MAPK, and AP-1, leading to a decrease in cell viability and activation of caspase-3 activity in HCFs. These results advance additional insights into the mechanisms of S1P-initiated inflammatory responses and proapoptotic effect through COX-2/PGE_2_ upregulation in HCFs.

## 2. Materials and Methods

### 2.1. Reagents and Antibodies

Fetal bovine serum (FBS), DMEM/F-12 medium, PLUS-Lipofectamine, and TRIzol reagent were purchased from Invitrogen (Carlsbad, CA, USA). The Western blotting detection system, enhanced chemiluminescence (ECL), and Hybond C membrane were purchased from GE Healthcare Biosciences (Buckinghamshire, UK). COX-2 antibody was from Abcam (Cambridge, UK). Phospho-JNK1/2 (#4668, Thr^183^/Tyr^185^), phospho-p38 MAPK (#9211, Thr^180^/Tyr^182^), phospho-p42/p44 MAPK (#9101, Thr^202^/Tyr^204^), phospho-EGFR (#4407, Tyr^1173^), phospho-Akt (#9271, Ser^473^), c-Jun (#9165), and PI3K p110*α* (#4249) antibodies were from Cell Signaling (Danvers, MA, USA). EGFR (sc-03-G), MMP9 (sc-10737), Akt (sc-1619), caspase-3 (sc-22140), and phospho-c-Jun (sc-822, Ser^63^) antibodies were from Santa Cruz (Santa Cruz, CA, USA). CAY10444, W123, and sphingosine 1-phosphate (S1P) were from Cayman (Ann Arbor, MI, USA). Pertussis toxin (PTX), GPA2A, SP600125, SB202190, PD98059, AG1478, LY294002, and tanshinone IIA were from Biomol (Plymouth Meeting, PA, USA). The bicinchoninic acid (BCA) protein assay kit was purchased from Pierce (Rockford, IL, USA). Glyceraldehyde 3-phosphate dehydrogenase (GAPDH) antibody was purchased from Biogenesis (Bournemouth, UK). siRNAs for c-Jun (HSS180003, HSS105641, and HSS105642) were from Invitrogen (Carlsbad, CA, USA). Enzymes, siRNAs for Akt (SASI_Hs01_00205545), EGFR (SASI_Hs01_00215449), p110 (SASI_Hs01_00219339), and MMP9 (SASI_Hs02_00338726), MMP2/9 inhibitor, CRM197, 2,3-bis-(2-methoxy-4-nitro-5-sulfophenyl)-2H-tetrazolium-5-carboxanilide (XTT) assay kit, and other chemicals were from Sigma (St. Louis, MO, USA).

### 2.2. Cell Culture and Treatment

HCFs were obtained from ScienCell Research Laboratories (San Diego, CA, USA). The procedures of cell culture were performed as previously described [15]. Experiments of treatment with S1P and inhibitors were conducted with cell passages 4 to 7.

### 2.3. Preparation of Cell Extracts and Western Blot Analysis

HCFs were exposed to S1P at 37°C for the designed time intervals. As previously described [10], the whole-cell extract was yielded with ice-cold PBS washing, scraping, and centrifugation at 45,000 × *g* for 1 h at 4°C. Procedures of Western blot analysis were conducted as previously described [15]. After being transferred, membranes were incubated with a specific antibody (1 : 1000) overnight, the membranes were washed four times for 5 min each, and the membranes were incubated with an anti-rabbit horseradish peroxidase antibody of a 1 : 2000 dilution for 1 h. The immunoreactive bands were detected by ECL reagents, and UN-SCAN-IT gel version 6.1 (Orem, Utah, USA) was applied to quantify the densitometry of bands normalized to GAPDH. Each experiment was repeated in three individual experiments (*n* = 3).

### 2.4. MMP Gelatin Zymography

HCFs were exposed to S1P at 37°C for the designed time intervals. The culture media were saved for this assay. Cells and debris have been removed by centrifugation at 1000 × *g* at 4°C for 10 min. As previously described [24], these samples were electrophoretically separated on 10% SDS-PAGE copolymerized with 1 mg/ml gelatin under nonreducing conditions (Sigma-Aldrich, St. Louis, MS, USA). The proform zymogens were quantified because the active form of MMPs was not reliably detectable in this study.

### 2.5. Total RNA Extraction and PCR/Real-Time PCR Analysis

Total RNA was isolated using TRIzol from HCFs treated with S1P for the designed time intervals. As previously described [25], total RNA extraction and PCR/real-time PCR were performed. Based on Genbank entries for human COX-2 and GAPDH, oligonucleotide primers were designed. For amplification of DNA, the following primers were used: COX-2 (NM_000963.4; product length: 146 bp; Tm: 60-61°C): 5′-TGCATTCTTTGCCCAGCACT-3′ (sense), 5′-AAAGGCGCAGTTTACGCTGT-3′ (antisense); GAPDH (NM_001357943.2; product length: 170 bp; Tm: 55-57°C): 5′-CGAGATCCCTCCAAAATCAA-3′ (sense), 5′-TTCACACCCATGACGAACAT-3′ (antisense).

Real-time PCR was performed using the method of the TaqMan gene expression assay system as previously described [25], and mixes of probe and primers for COX-2 and endogenous GAPDH control genes were applied. The real-time PCR assay was performed using a 7500 Real Time-PCR System (Applied Biosystems, Foster City, CA, USA). Relative gene expression was determined by the *ΔΔ*Ct method, where Ct meant the threshold cycle.

### 2.6. Plasmid Construction, Transfection, and Luciferase Reporter Gene Assays

For the construction of COX-2-Luc plasmids, a human COX-2 promoter located within a region spanning from -483 to +37 bp was cloned into the pGL3-basic vector (Promega, Madison, WI, USA). A series of point mutations were introduced into the AP-1 binding site of the COX-2 promoter by mismatched primer mutation PCR, as previously described [25]. To prepare the plasmids, QIAGEN plasmid DNA preparation kits were used, according to the instructions of the manufacturer, using the Lipofectamine reagent to transfect the plasmids into HCFs. Whole-cell lysates were used to determine the COX-2-Luc activity according to the instructions of the manufacturer (Promega, Madison, WI, USA). Firefly luciferase activities were standardized for *β*-galactosidase activity.

### 2.7. Transient Transfection with siRNAs

HCFs cells were plated in 12-well culture plates at 3 × 10^5^ cells/ml (1 ml/well) for 24 h incubation, reaching about 80% confluence. After being washed once with PBS and once with serum-free DMEM/F-12, cells were incubated with 0.4 ml of serum-free DMEM/F-12 medium each well. According to the instructions of the manufacturer, the siRNA was prepared using the Lipofectamine 2000 transfection reagent and transiently transfected.

### 2.8. Chromatin Immunoprecipitation (ChIP) Assay

ChIP analysis was conducted as previously described [26], to detect the association of nuclear proteins with human COX-2 promoters. In brief, for cross-link chromatin, HCFs were incubated with 1% formaldehyde for 10 min at 37°C. According to the manufacturer's instructions, after being washed thrice with ice-cold PBS containing 1 mM phenylmethylsulfonyl fluoride (PMSF) and 1% aprotinin, the pellet was used to prepare the soluble chromatin by using a ChIP assay kit (Upstate, Essex County, NY, USA). Then, the soluble chromatin was immunoprecipitated without (control) or with an anti-c-Jun antibody and normal goat immunoglobulin G (IgG). For avoiding the possibility of amplification artifacts, PCR products for all SYBR Green primer pairs were verified to produce single products by a high-resolution melt curve. The relative gene levels were calculated using the comparative Ct method (∆∆Ct). The DNA was resuspended in H_2_O and subjected to PCR amplification with the AP1 primers (AF044206.1; product length: 168 bp; Tm: 59°C): F: 5′-CACCGGGCTTACGCAATT TT-3′ and R: 5′-ACGCTCACTGCAAGTCGTAT-3′.

### 2.9. Measurement of PGE_2_ Release

The serum-free cells were exposed to S1P for the indicated time intervals. The conditioned medium was saved and applied to measure PGE_2_ levels using an EIA kit as specified by the manufacturer (Cayman Chemicals).

### 2.10. Cell Viability Assay

Cell viability and proliferation analysis were determined by using an XTT assay kit according to the manufacturer's instructions (https://www.sigmaaldrich.com/technical-documents/protocols/biology/roche/cell-proliferation-kit-xtt-assay.html).

### 2.11. Statistical Analysis of Data

This study applied GraphPad Prism Program 6.0 software (GraphPad, San Diego, CA, USA) to perform statistical analysis. All the data were expressed as the mean ± SEM, for at least three individual experiments (*n* = number of independent cell culture preparations). Data were analyzed by one-way ANOVA followed by Tukey's post hoc test when comparing more than two groups of data. *P* values of 0.05 were considered to be statistically significant.

## 3. Results

### 3.1. S1P-Induced COX-2 Expression Is Regulated by EGFR Transactivation

**Figure 1:**
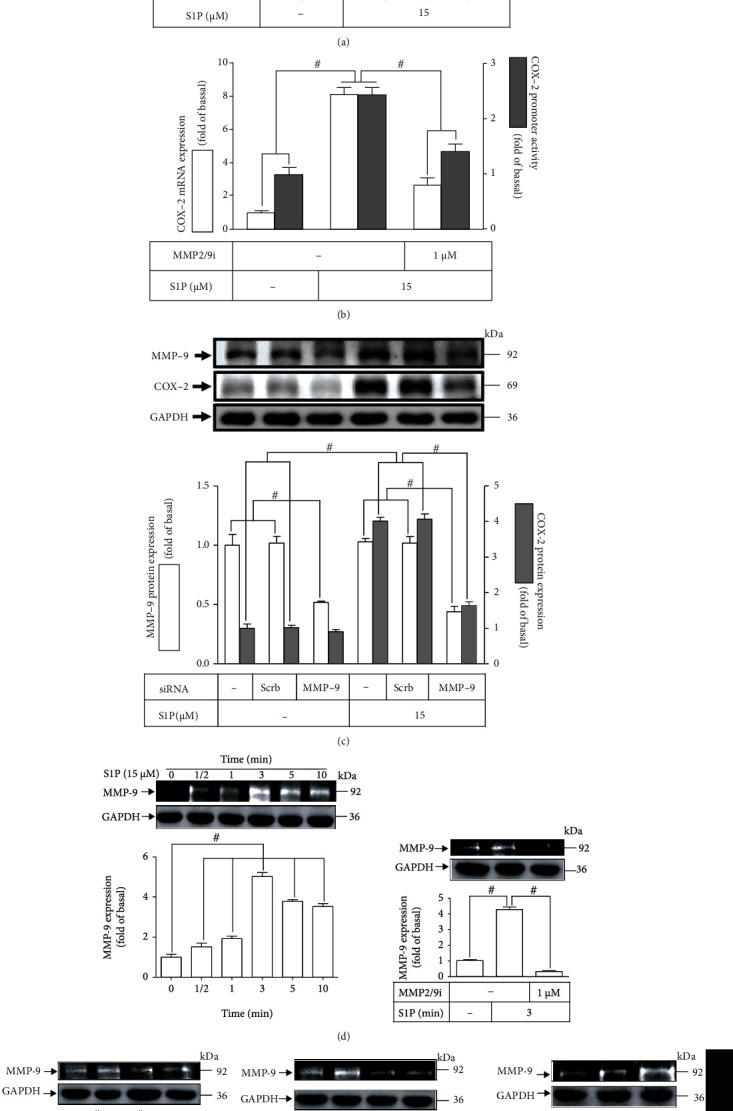
S1P induces COX-2 expression via MMP9 activity in HCFs. (a) Cells were pretreated with MMP2/9 inhibitor (MMP2/9i; 0.01, 0.1, and 1 *μ*M) for 1 h and then incubated with 15 *μ*M S1P for 8 h. The levels of COX-2 and GAPDH used as an internal control were analyzed by Western blot. (b) Cells were transfected with COX-2 promoter-luciferase reporter gene, pretreated without or with MMP2/9i (1 *μ*M) for 1 h, and then incubated with 15 *μ*M S1P for 4 h (mRNA level) or 1 h (promoter activity). The COX-2 mRNA and promoter activity were analyzed by real-time PCR (open bar) and promoter assay (gray bar). (c) Cells were transfected with scrambled or MMP9 siRNA for 24 h and then exposed to 15 *μ*M S1P for 8 h. The levels of COX-2, GAPDH, and MMP9 proteins were analyzed by Western blot. (d) Cells were pretreated with or without 1 *μ*M MMP2/9 inhibitor for 1 h and then incubated with 15 *μ*M S1P for 0.5, 1, 3, 5, and 10 min. (e) Cells were pretreated with or without 10 *μ*M W123, 10 *μ*M CAY10444, 10 *μ*M GPA2A, 100 ng/ml PTX, or 10 *μ*g/ml CRM197 for 1 h and then incubated with 15 *μ*M S1P for 3 min. Cell lysates and medium were collected to determine the levels of MMP9 and GAPDH by gelatin zymography and Western blot, respectively. Data are expressed as the mean ± SEM of three individual experiments (*n* = 3). ^#^*P* < 0.05, as compared with the control or pretreatment with inhibitor indicated in the figure.

GPCR-mediated transactivation of growth factor receptors contributes to many kinds of pathogenesis including inflammatory responses [27–29]. MMP9 is recognized as an extracellular protease participating in the crosstalk between GPCR and RTK signaling pathway. S1PRs in vascular smooth muscle cells have been revealed to transactivate the platelet-derived growth factor receptor (PDGFR) and EGFR [30, 31]. Here, we determined whether S1PR-mediated EGFR transactivation is implicated in increased COX-2 expression by S1P. HCFs were received with MMP2/9 inhibitor pretreatment and then challenged with S1P for the indicated time intervals to evaluate the effect of MMP9 on the COX-2 level stimulated by S1P. Data in Figure 1(a) showed that MMP2/9 inhibitor concentration-dependently mitigated the protein level of COX-2 triggered by S1P, to further examine whether MMP9 activity regulates S1P-initiated *cox-2* gene expression which was assessed by the promoter activity assay and real-time PCR. Our data showed that S1P-induced COX-2 promoter activity and *cox-2* mRNA expression were significantly blocked by MMP2/9 inhibitor pretreatment (Figure 1(b)). We further used MMP9 siRNA to verify the role of MMP9 on the COX-2 upregulation produced by S1P in HCFs. As displayed in Figure 1(c), in HCFs, transfection with MMP9 siRNA downregulated the protein level of MMP9 and abrogated the S1P-triggered COX-2 protein level as compared with that of scrambled siRNA transfection. We also revealed that S1P time-dependently promoted MMP9 activity with a maximal response within 3 min and the response sustained for 10 min (Figure 1(d)). Moreover, the results of gelatin zymography showed that MMP2/9 inhibitor pretreatment significantly attenuated the activity of MMP9 (Figure 1(d)). We further determined whether S1P receptor subtypes and G proteins were involved in the enhanced MMP9 activity; HCFs were pretreated with S1PR1 antagonist (W123), S1PR3 antagonist (CAY10444), G_q_ protein antagonist (GPA2A), G_i_ antagonist (pertussis toxin (PTX)), or the inhibitor of HB-EGF (CRM197) and then challenged with S1P for 3 min. The cultured media were saved to be analyzed by gelatin zymography to determine MMP9 levels. The findings showed that pretreatment with W123, CAY10444, GPA2A, or PTX reduced MMP9 activity induced by S1P, but not with CRM197 in HCFs (Figure 1(e)). These results suggested that the increased level of COX-2 triggered by S1P is mediated through SIPR1/3 coupled to G_q_ or G_i_ protein-dependent activation of MMP9 in HCFs.

**Figure 2:**
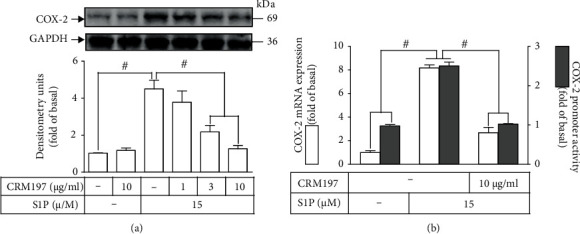
HB-EGF is involved in S1P-induced COX-2 expression in HCFs. (a) Cells were pretreated without or with CRM197 (1, 3, and 10 *μ*g/ml) for 1 h and then incubated with 15 *μ*M S1P for 8 h. The levels of COX-2 and GAPDH used as an internal control were analyzed by Western blot. (b) Cells were transfected with COX-2 promoter-luciferase reporter gene, pretreated with CRM197 (10 *μ*g/ml) for 1 h, and then incubated with 15 *μ*M S1P for 4 h (mRNA level) or 1 h (promoter activity). The COX-2 mRNA and promoter activity were analyzed by real-time PCR (open bar) and promoter assay (gray bar). Data are expressed as the mean ± SEM of three individual experiments (*n* = 3). ^#^*P* < 0.05, as compared with the control or pretreatment with inhibitor indicated in the figure.

To determine whether MMP9-mediated EGFR transactivation via cleavage of HB-EGF participates in the COX-2 upregulation stimulated by S1P, an inhibitor of HB-EGF (CRM197) was used for this purpose. The result in Figure 2(a) showed that in HCFs, S1P-produced COX-2 protein upregulation was concentration-dependently attenuated by CRM197 pretreatment. Further, both promoter activity assay and real-time PCR were performed to verify whether S1P-enhanced gene expression of COX-2 is regulated by HB-EGF. The data demonstrated that CRM197 pretreatment significantly inhibited S1P-induced COX-2 promoter activity and mRNA expression of COX-2 (Figure 2(b)). These results implied that the increased level of COX-2 induced by S1P is mediated through SIPR1/3-dependent transactivation of EGFR by HB-EGF in HCFs.

### 3.2. Involvement of EGFR in S1P-Induced COX-2 Expression

**Figure 3:**
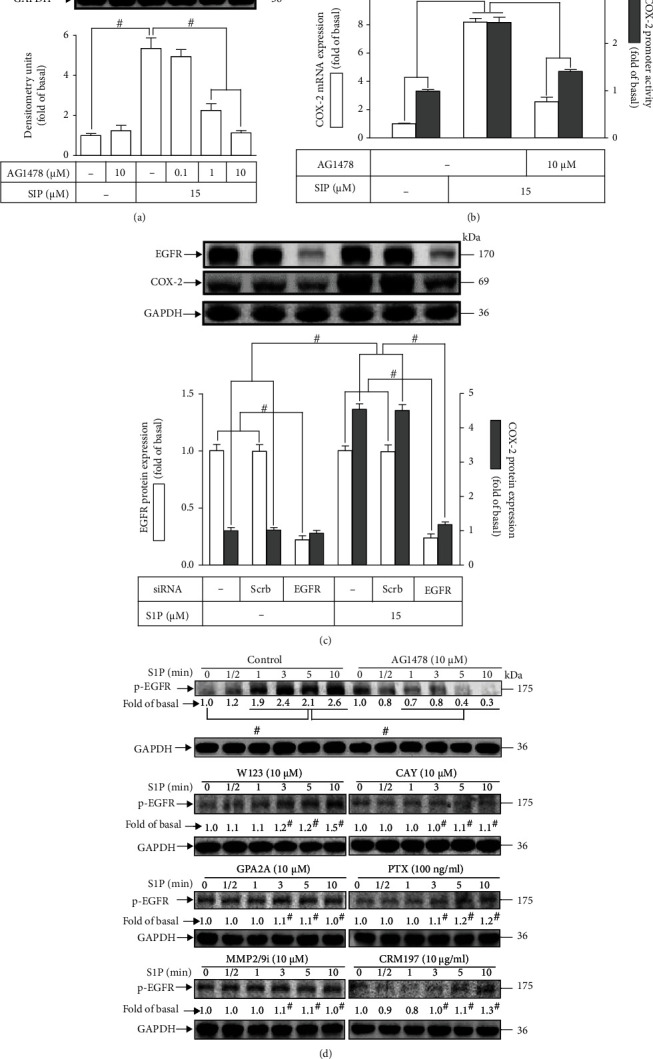
S1P-induced EGFR transactivation mediates COX-2 expression in HCFs. (a) Cells were pretreated with AG1478 (0.1, 1, and 10 *μ*M) for 1 h and then incubated with 15 *μ*M S1P for 8 h. The levels of COX-2 and GAPDH used as an internal control were analyzed by Western blot. (b) Cells were transfected with COX-2 promoter-luciferase reporter gene, pretreated without or with AG1478 (10 *μ*M) for 1 h, and then incubated with 15 *μ*M S1P for 4 h (mRNA level) or 1 h (promoter activity). The COX-2 mRNA and promoter activity were analyzed by real-time PCR (open bar) and promoter assay (gray bar), respectively. (c) Cells were transfected with scrambled or EGFR siRNA for 24 h and then exposed to 15 *μ*M S1P for 8 h. The levels of COX-2, GAPDH, and EGFR proteins were analyzed by Western blot. (d) Cells were pretreated without or with 10 *μ*M AG1478, 10 *μ*M W123, 10 *μ*M CAY10444, 10 *μ*M GPA2A, 100 ng/ml PTX, 10 *μ*M MMP2/9 inhibitor, or 10 *μ*g/ml CRM197 for 1 h and then incubated with 15 *μ*M S1P for 0.5, 1, 3, 5, and 10 min. The levels of phospho-EGFR and GAPDH used as an internal control were analyzed by Western blot. Data are expressed as mean ± SEM of three individual experiments (*n* = 3). ^#^*P* < 0.05, as compared with the control or pretreatment with inhibitor indicated in the figure.

EGFR possesses several effects contributing to the regulation of cellular functions including differentiation, cell growth, and development [32, 33]. EGFR expressed in various types of cells has also been demonstrated to regulate the expression of inflammatory proteins [27, 31, 34, 35]. Hence, we investigated the role of EGFR in the S1P-induced responses; an EGFR inhibitor AG1478 was used for this purpose. The findings presented in Figure 3(a) showed that the S1P-prompted COX-2 protein induction was concentration-dependently inhibited by AG1478 pretreatment. Moreover, AG1478 pretreatment also mitigated COX-2 promoter activity as well as *cox-2* mRNA expression triggered by S1P (Figure 3(b)), suggesting that in HCFs, EGFR acts an important role in the S1P-prompted *cox-2* gene expression. Transfection of HCFs with EGFR siRNA further addressed the role of EGFR in the S1P-triggered COX-2 expression. As presented in Figure 3(c), EGFR siRNA transfection significantly downregulated EGFR protein expression, which also led to a decrease in the S1P-enhanced COX-2 expression. Furthermore, the levels of EGFR phosphorylation were examined by Western blot to determine whether S1P-induced responses required the activation of EGFR. The findings presented in Figure 3(d) demonstrated that S1P time-dependently promoted the levels of EGFR phosphorylation, an active status, with a maximal response within 5 min, which was markedly attenuated by pretreatment with an EGFR inhibitor AG1478 during the period of observation. Additionally, pretreatment with CAY (10 *μ*M), W123 (10 *μ*M), PTX (100 ng/ml), MMP2/9 inhibitor (10 *μ*M), GPA2A (10 *μ*M), or CRM197 (10 *μ*M) also attenuated the S1P-stimulated levels of phospho-EGFR (Figure 3(d)). These data implied that S1P-activated S1PR1/3 coupled to G_i_ or G_q_ protein-mediated EGFR transactivation via HB-EGF-stimulated EGFR phosphorylation, leading to upregulation of COX-2 in HCFs.

### 3.3. PI3K/Akt Is Involved in S1P-Induced COX-2 Expression

**Figure 4:**
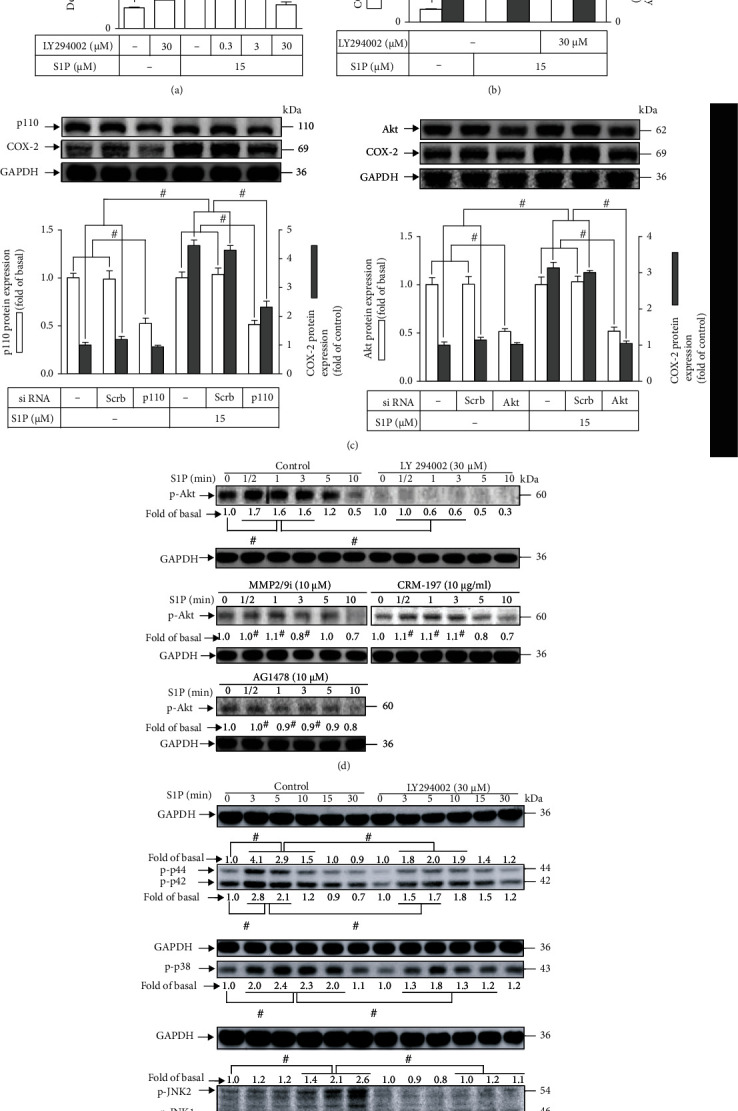
S1P-induced COX-2 expression is mediated through PI3K/Akt in HCFs. (a) Cells were pretreated without or with LY294002 (0.3, 3, and 30 *μ*M) for 1 h and then incubated with 15 *μ*M S1P for 8 h. The levels of COX-2 and GAPDH used as an internal control were analyzed by Western blot. (b) Cells were transfected without or with COX-2 promoter-luciferase reporter gene, pretreated with LY294002 (30 *μ*M) for 1 h, and then incubated with 15 *μ*M S1P for 4 h (mRNA level) or 1 h (promoter activity). The COX-2 mRNA and promoter activity were analyzed by real-time PCR (open bar) and promoter assay (gray bar). (c) Cells were transfected with scrambled, p110 (left panel), or Akt (right panel) siRNA for 24 h and then exposed to 15 *μ*M S1P for 8 h. The levels of COX-2, GAPDH, p110, and Akt proteins were analyzed by Western blot. (d) Cells were pretreated with or without 30 *μ*M LY294002, 10 *μ*M MMP2/9 inhibitor, 10 *μ*g/ml CRM197, or 10 *μ*M AG1478 for 1 h and then incubated with 15 *μ*M S1P for 0.5, 1, 3, 5, and 10 min. The levels of phospho-Akt and GAPDH were determined by Western blot. (e) Cells were pretreated without or with 30 *μ*M LY294002 for 1 h and then incubated with 15 *μ*M S1P for 0, 3, 5, 10, 15, and 30 min. The levels of phospho-p44/p42, phospho-p38, phospho-JNK1/2, and GAPDH were determined by Western blot. Data are expressed as the mean ± SEM of three individual experiments (*n* = 3). ^#^*P* < 0.05, as compared with the control or pretreatment with inhibitor indicated in the figure.

S1P is able to modulate the intracellular functions of HCFs via activation of PI3K/Akt [10, 34]. A PI3K inhibitor LY294002 was used to investigate the function of PI3K/Akt in the induction of COX-2 stimulated by S1P in HCFs. Data presented in Figure 4(a) demonstrated that LY294002 pretreatment dose-dependently attenuated the expression of S1P-promoted COX-2. Additionally, pretreatment with LY294002 attenuated the COX-2 promoter activity as well as *cox-2* mRNA expression induced by S1P (Figure 4(b)), implying that in HCFs, the S1P-triggered *cox-2* gene upregulation is dependent on PI3K/Akt cascade. We further verified the roles of PI3K/Akt in the expression of COX-2; as presented in Figure 4(c), downregulation of p110 or Akt protein level by transfection of HCFs with either p110 or Akt siRNA significantly attenuated the COX-2 protein level triggered by S1P. Besides, to dissect whether the phosphorylation status of Akt activation was indispensable for S1P-triggered responses, the level of phospho-Akt was analyzed by the Western blot assay using a phospho-Akt antibody. As presented in Figure 4(d), S1P progressively boosted Akt phosphorylation with a maximal level within 0.5-3 min during the period of observation, which was markedly mitigated by pretreatment with LY294002. In addition, pretreatment with MMP2/9 inhibitor (10 *μ*M), CRM197 (10 *μ*M), or AG1478 (10 *μ*M) also attenuated the S1P-enhanced levels of phospho-Akt. These data implied that S1P-stimulated Akt phosphorylation leading to COX-2 upregulation is caused by the MMP9-mediated HB-EGF-dependent EGFR pathway in HCFs.

PI3K has been revealed to regulate the MAPK pathway leading to the overexpression of target proteins in many kinds of cells [10, 27, 34, 36]. To examine whether PI3K can activate MAPKs in response to S1P stimulation, HCFs preincubated with LY294002 for 1 h were challenged with S1P for the indicated time intervals. The data in Figure 4(e) demonstrated that pretreatment with LY294002 could attenuate with the S1P-stimulated phosphorylation level of JNK1/2, p38 MAPK, and p44/p42 MAPK. These findings suggested that PI3K is an upstream component involved in the S1P-stimulated phosphorylation of MAPKs in HCFs.

### 3.4. AP-1 Is Required for S1P-Induced COX-2 Expression

**Figure 5:**
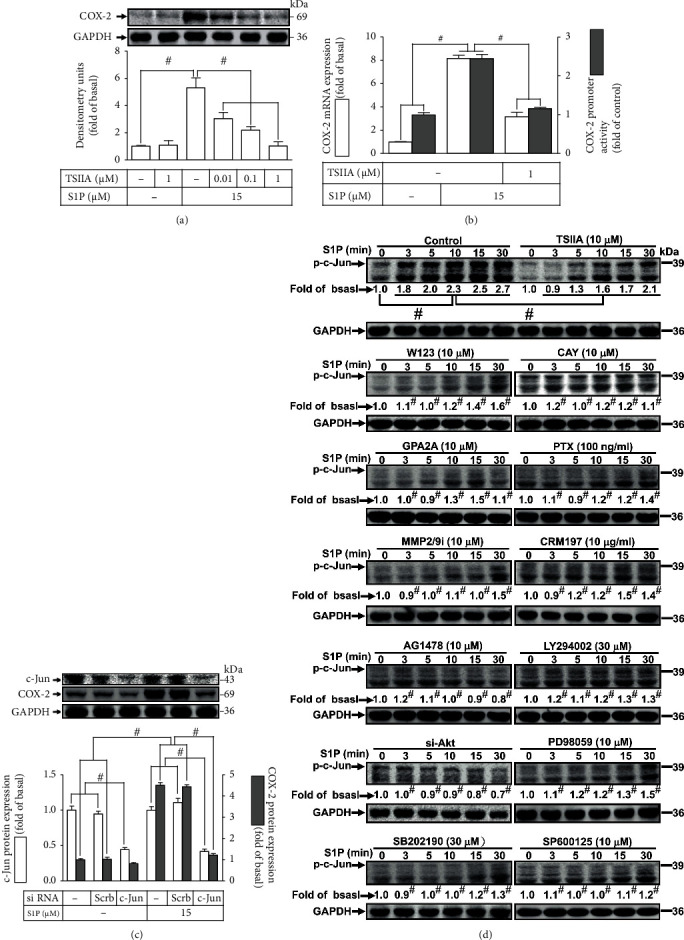
Involvement of c-Jun in S1P-induced COX-2 expression in HCFs. (a) Cells were pretreated without or with tanshinone IIA (TSIIA; 0.01, 0.1, and 1 *μ*M) for 1 h and then incubated with 15 *μ*M S1P for 8 h. The levels of COX-2 and GAPDH used as an internal control were analyzed by Western blot. (b) Cells were transfected without or with COX-2 promoter-luciferase reporter gene, pretreated with TSIIA (1 *μ*M) for 1 h, and then incubated with 15 *μ*M S1P for 4 h (mRNA level) or 1 h (promoter activity). The COX-2 mRNA and promoter activity were analyzed by real-time PCR (open bar) and promoter assay (gray bar), respectively. (c) Cells were transfected with scrambled or c-Jun siRNA for 24 h and then exposed to 15 *μ*M S1P for 8 h. The levels of COX-2, GAPDH, and c-Jun proteins were analyzed by Western blot. (d) Cells were pretreated without or with 10 *μ*M TSIIA, 10 *μ*M W123, 10 *μ*M CAY, 10 *μ*M GPA2A, 100 ng/ml (PTX), 10 *μ*M MMP2/9i, 10 *μ*g/ml CRM197, 10 *μ*M AG1478, 30 *μ*M LY294002, 10 *μ*M PD98059, 30 *μ*M SB202190, or 10 *μ*M SP600125 for 1 h or transfected with scrambled or Akt siRNA, respectively, and then incubated with 15 *μ*M S1P for 0, 3, 5, 10, 15, and 30 min. The levels of phospho-c-Jun and GAPDH were determined by Western blot. Data are expressed as the mean ± SEM of three individual experiments (*n* = 3). ^#^*P* < 0.05, as compared with the control or pretreatment with inhibitor indicated in the figure.

S1P could modulate a wide array of intracellular functions in many kinds of cells via activating AP-1 signaling [12, 34]. To verify activation of AP-1 is required for S1P-upregulated COX-2; HCFs were preincubated with tanshinone IIA (TSIIA), a selective AP-1 inhibitor, which attenuated the S1P-upregulated COX-2 protein level (Figure 5(a)), mRNA expression, and promoter activity (Figure 5(b)). To further prove that c-Jun (an AP-1 subunit) is indispensable for the S1P-triggered COX-2 expression, as presented in Figure 5(c), transfection with c-Jun siRNA significantly downregulated the c-Jun protein level and reduced the COX-2 expression induced by S1P. Moreover, to determine whether phosphorylation of c-Jun is required in S1P-triggered responses, as demonstrated in Figure 5(d), S1P progressively promoted an increased level of phospho-c-Jun reaching a maximal response within 10-30 min, which was reduced by TSIIA pretreatment. Moreover, the level of phospho-c-Jun was also mitigated by pretreatment with CAY10444, W123, GPA2A, PTX, MMP2/9i, CRM197, AG1478, LY294002, Akt siRNA, SP600125, SB202190, or PD98059 (Figure 5(d)). These results suggested that in HCFs, S1P-activated c-Jun activity is regulated by S1PR1/3, G_i_, G_q_, MMP9, HB-EGF, EGFR, PI3K/Akt, JNK1/2, p42/p44 MAPK, and p38 MAPK, resulting in COX-2 upregulation.

### 3.5. Involvement of c-Jun in *cox-2* Gene Promoter Activity Induced by S1P

**Figure 6:**
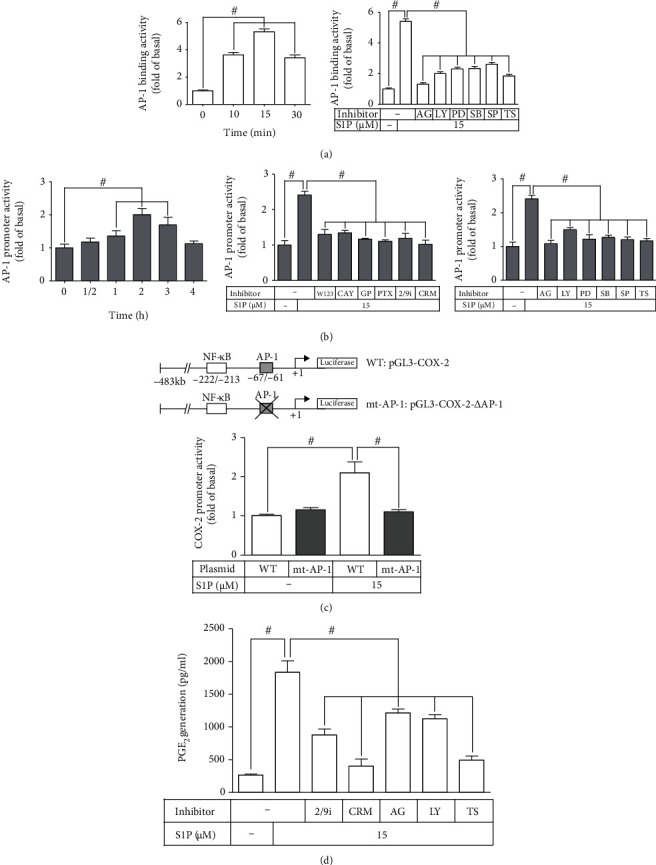
COX-2 promoter activity is stimulated by S1P mediated through an AP-1-dependent pathway. (a) Cells were incubated with S1P for the indicated time intervals (0, 10, 15, and 30 min; left panel). Cells were pretreated without or with AG1478 (10 *μ*M), LY294002 (30 *μ*M), PD98059 (10 *μ*M), SB202190 (30 *μ*M), or SP600125 (10 *μ*M) for 1 h and then incubated with S1P for 15 min (right panel). The binding activity of phospho-c-Jun and promoter was analyzed by a ChIP assay (*n* = 3), as described in Materials and Methods. (b) Cells were transfected with an AP-1-luciferase reporter gene and then incubated with S1P for 0, 0.5, 1, 2, 3, and 4 h (left panel) and pretreated with W123 (10 *μ*M), CAY (10 *μ*M), GPA2A (10 *μ*M), PTX (100 ng/ml), MMP2/9i (10 *μ*M), or CRM197 (10 *μ*g/ml) (middle panel) and AG1478 (10 *μ*M), LY294002 (30 *μ*M), PD98059 (10 *μ*M), SB202190 (30 *μ*M), SP600125 (10 *μ*M), or tanshinone IIA (10 *μ*M) (right panel) for 1 h and then incubated with S1P for 2 h. (c) The schematic picture represented two different 5′-promoter regions of COX-2 promoter constructs, both wild type (WT) and mt-AP-1 modified by single-point mutation of the AP-1 binding site fused into the pGL-luciferase reporter gene. WT COX-2 promoter-reporter gene (WT-COX-2) or AP-1 mutated COX-2 promoter-reporter gene (mt-AP-1-COX-2) were transfected into cells and then incubated without or with S1P for 1 h. The promoter-reporter activity was determined in the cell lysates. (d) Cells were pretreated without or with MMP2/9i, CRM197, AG1478, LY294002, or tanshinone IIA for 1 h and then incubated with S1P for 8 h. The levels of PGE_2_ were analyzed by EIA. Data are expressed as the mean ± SEM of three individual experiments (*n* = 3). ^#^*P* < 0.05, as compared with the control or pretreatment with inhibitor indicated in the figure.

We have addressed that in HCFs, S1P promotes phosphorylation of c-Jun related to COX-2 expression. Next, to examine whether activated c-Jun is able to connect to the promoter sequence of the *cox-2* gene, we performed a ChIP-PCR analysis. As presented in Figure 6(a) (left panel), S1P time-dependently promoted the promoter binding activity of c-Jun which was mitigated by AG1478, LY294002, PD98059, SB202190, SP600125, or tanshinone IIA pretreatment (right panel). These findings implied that S1P-promoted c-Jun binding activity is caused by EGFR, PI3K/Akt, JNK1/2, p44/p42 MAPK, and p38 MAPK-dependent pathways. We further dissected whether AP-1 activation was essential for the gene expression of *cox-2* triggered by S1P. We used a luciferase activity assay with an AP-1 binding site within the promoter sequence to analyze the activity of c-Jun in transcription. The data in Figure 6(b) demonstrated that S1P time-dependently boosted AP-1 promoter activity reaching a maximal response within 2 h (left panel), which was significantly attenuated by CAY, GPA2A, PTX, W123, MMP2/9i, CRM197, AG1478, LY294002, PD98059, SB202190, SP600125, or tanshinone IIA pretreatment (middle and right panels). These results indicated that S1P-enhanced transcription activity of c-Jun is through a PI3K/Akt/MAPKs (p44/p42 MAPK, JNK1/2, and p38 MAPK) cascade activated by S1PR1/3-dependent transactivation of EGFR. Additionally, to further ensure that c-Jun contributes to the promoter activity of COX-2 triggered by S1P mediated through binding to its regulatory site on the promoter region of COX-2, we constructed both the mt-AP-1 promoter of COX-2 mutated by a single-point mutation on the c-Jun binding site and wild type (WT) (Figure 6(c), upper panel). As presented in the lower panel of Figure 6(d), HCFs transfected with mt-AP-1-plasmid of COX-2 promoter significantly blocked the promoter activity of COX-2 stimulated by S1P as compared with that of WT AP-1 promoter of COX-2, indicating that the promoter activity of COX-2 triggered by S1P is dependent on c-Jun (AP-1 subunit). These results directly proved that the S1P-triggered promoter activity of COX-2 is caused by increasing the binding of AP-1 on the promoter region of COX-2 in HCFs.

To further dissect whether S1P-increased protein levels of COX-2 contribute to a parallel of PGE_2_ generation, we collected the cultured media and used an ELISA kit to measure PGE_2_ levels. These findings displayed that S1P-promoted PGE_2_ generation was attenuated by MMP2/9i, CRM197, AG1478, LY294002, or tanshinone IIA pretreatment, implying that S1P-induced PGE_2_ generation is dependent on COX-2 and mediated through EGFR transactivation-dependent PI3K/Akt/AP-1 pathway in HCFs (Figure 6(d)). These results implied that in HCFs, S1P-enhanced COX-2 upregulation is mediated through the S1PR1/3-mediated EGFR transactivation to activate PI3K/Akt/MAPKs (JNK1/2, p38 MAPK, and p44/p42 MAPK) cascade-dependent AP-1 activity leading to *cox-2* gene transcription, which in turn boosts the production of PGE_2_.

### 3.6. The Involvement of COX-2/PGE_2_ in S1P-Induced Cell Apoptosis

**Figure 7:**
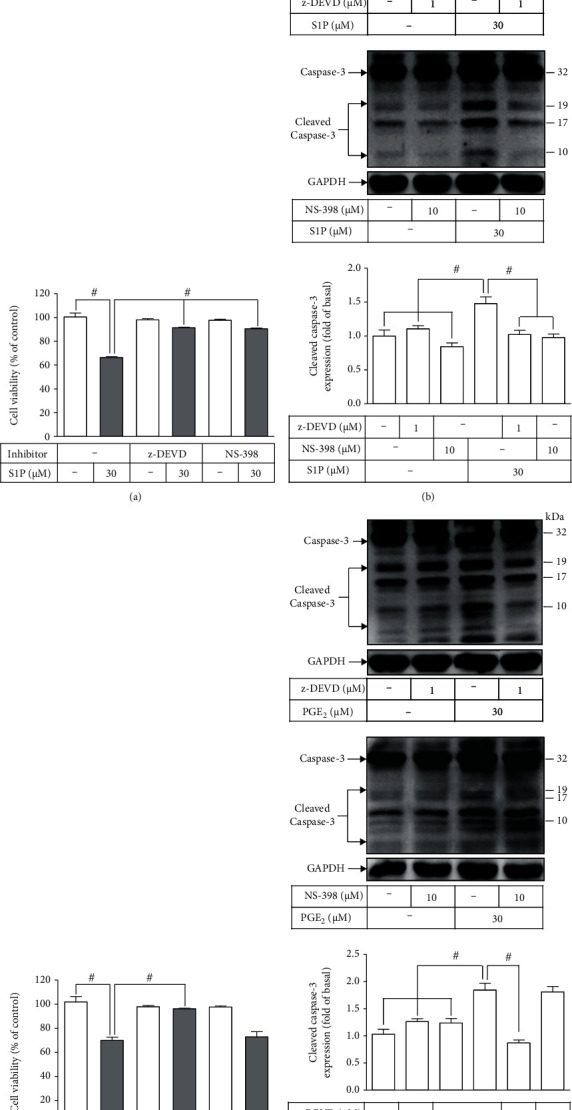
S1P-induced COX-2/PGE_2_ stimulates apoptotic caspase-3 activation. (a) Cells were pretreated without or with z-DEVD (3 *μ*M) or NS-398 (10 *μ*M) for 1 h and then incubated with 30 *μ*M S1P for 24 h. The cell viability was determined by an XTT assay. (b) Cells were pretreated without or with z-DEVD (3 *μ*M; upper panel) or NS-398 (10 *μ*M; lower panel) and then incubated with 30 *μ*M S1P for 8 h, and then, whole-cell lysates were analyzed to determine the levels of the cleaved form of caspase-3 and GAPDH used as an internal control by Western blot. (c) Cells were pretreated without or with z-DEVD (3 *μ*M) or NS-398 (10 *μ*M) for 1 h and then incubated with 30 *μ*M PGE_2_ for 24 h. The cell viability was determined by an XTT assay. (d) Cells were pretreated without or with z-DEVD (3 *μ*M; upper panel) or NS-398 (10 *μ*M; lower panel) for 1 h and then incubated with 30 *μ*M PGE_2_ for 12 h, and then, whole-cell lysates were analyzed to determine the levels of the cleaved form of caspase-3 and GAPDH used as an internal control by Western blot. Data are expressed as the mean ± SEM of three individual experiments (*n* = 3). ^#^*P* < 0.05, as compared with the control or pretreatment with inhibitor indicated in the figure.

S1P has been found to trigger cell apoptosis in mesangial cells. [22]. Hence, to differentiate the role of COX-2 in the S1P-stimulated cell apoptosis via activation of caspase-3, both the COX-2 inhibitor NS-398 and caspase-3 inhibitor z-DEVD were used for these purposes. As shown in Figure 7(a), exposure of HCFs to S1P (30 *μ*M) reduced cell viability by about 40% as compared with that of vehicle, which can be rescued by pretreatment with either z-DEVD or NS-398, implying that S1P-stimulated apoptosis was mediated through COX-2-dependent activation of the caspase-3 pathway. We further applied Western blot to examine the levels of the cleaved form of caspase-3 as a marker of apoptosis. These data presented in Figure 7(b) revealed that S1P-triggered expression of cleaved caspase-3 was inhibited by pretreatment with either z-DEVD or NS-398. Next, we determined the involvement of COX-2-mediated PGE_2_ synthesis in cell viability and the expression of cleaved caspase-3 induced by S1P. As shown in Figure 7(c), HCFs challenged with PGE_2_ (30 *μ*M) attenuated cell viability of HCFs, which was rescued by pretreatment with z-DEVD but not NS-398. In addition, HCFs challenged with PGE_2_ also caused increased production of the cleaved caspase-3, which was mitigated by z-DEVD pretreatment but not NS-398 (Figure 7(d)). These results suggested that S1P-triggered cytotoxicity and apoptosis are mediated through COX-2/PGE_2_-dependent caspase-3 activity in HCFs.

## 4. Discussion

**Figure 8:**
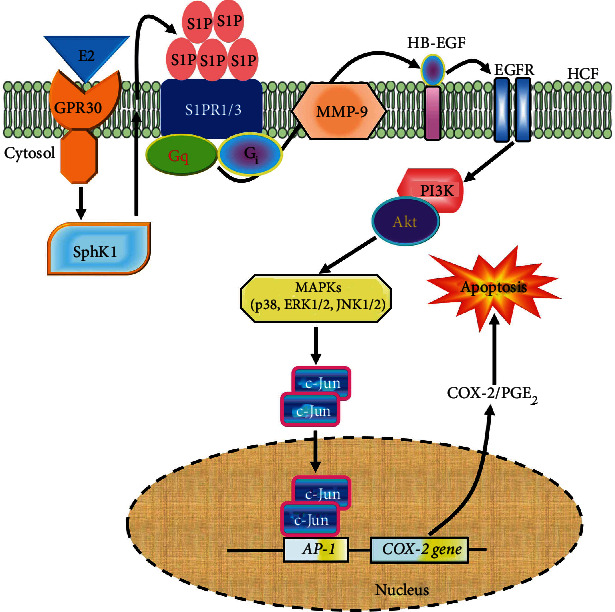
Schematic signaling pathways are involved in S1P-induced COX-2/PGE_2_ expression associated with activation of caspase-3 leading to apoptosis in HCFs. S1P could be upregulated mediated through estrogen/estrogen receptor-stimulated sphingosine kinase-1 (SphK1) activity [9], binding with S1PR1/3 coupled to G_q_ or G_i_ protein results in MMP9-mediated HB-EGF cleavage leading to activation of EGFR/PI3K/Akt/MAPKs (i.e., p42/p44 MAPK, p38 MAPK, and JNK1/2)-dependent AP-1 c-Jun. The COX-2 transcription and PGE_2_ generation are dependently regulated by an S1PR1/3-mediated EGFR transactivation to activate AP-1 binding activity. These signaling pathways contribute to the activation of AP-1 required for COX-2 expression and PGE_2_ generation in HCFs.

S1PR1-3 have been characterized to uniformly express in all chambers of myocardial tissues of human beings and rats [16]. S1P is metabolized from membrane structure sphingolipids and secreted into the extracellular environment in circulatory systems. It exerts several crucial functions via G protein-coupled S1PRs to regulate cell migration, differentiation, survival, and apoptosis [4]. However, in HCFs, the molecular mechanisms of how S1P stimulated the increase of COX-2 were not fully identified. Our results of the present study suggested that activation of S1PR1/3/G_i_ or G_q_-mediated EGFR transactivation-dependent PI3K/Akt/MAPKs (JNK1/2, p38 MAPK, and p42/p44 MAPK) pathway to activate AP-1 is necessary for the S1P-induced apoptosis mediated through *cox-2* gene upregulation and PGE_2_ production in HCFs (Figure 8).

GPCRs and RTKs are two major groups of membrane receptors that control multiple cellular functions via a wide range of intracellular signaling networks implicated in the pathophysiological processes. The first discovery of GPCR-EGFR crosstalk is involved in the progression of various cancers [28]. GPCRs have been revealed to transactivate EGFR activity mediated via both ligand-dependent and independent mechanisms [19]. GPCRs can cleave the membrane-bound EGFR-ligand precursors such as HB-EGF or directly activate the juxtamembrane tyrosine kinase domain of EGFR by intracellular signal molecules such as c-Src and Pyk2 [27, 29]. Moreover, estrogen-stimulated sphingosine kinase 1 activation leading to transactivation of the EGFR/MAPK system was also mediated through MMP and c-Src [30, 37]. Previous evidence has also indicated that estrogen signaling could contribute to COX-2 activation and PGI_2_ production protecting against atherosclerosis [38]. Therefore, there may be a crosstalk between estrogen and S1P in COX-2 expression modulating different cellular functions mediated through MMP/EGFR/MAPKs in various cardiovascular diseases. Our earlier study has demonstrated that S1P upregulates COX-2/PGE_2_ production via G protein-coupled S1PR1 and 3 activations in HCFs [15]. Here, we further revealed that S1P mediated through S1PR1 and 3 coupled to G_q_ or G_i_ protein via activation of MMP9 to induce COX-2 upregulation and PGE_2_ release. This hypothesis was supported by the evidence that (1) S1P-induced *cox-2* gene expression and COX-2 promoter activity were inhibited by pretreatment with MMP2/9 inhibitor or transfection with MMP9 siRNA; (2) S1P-stimulated MMP9 activity was inhibited by the antagonists of S1PR1 and 3, G_q_ and G_i_ inhibitors GPA2A and PTX, and MMP2/9 inhibitor, but not by CRM197; and (3) the transactivation of EGFR was triggered by HB-EGF in HCFs, which was attenuated by pretreatment with CRM197, an inhibitor of HB-EGF. Thus, GPCR-mediated transactivation of EGFR via MMP9/HB-EGF participates in S1P-induced COX-2 upregulation in HCFs. Earlier studies also indicated that S1P activates S1PRs, resulting in the MMP-dependent EGFR transactivation in MCF-7 cells [30]. In contrast, our previous study in human pulmonary alveolar epithelial cells revealed that S1P-activated AP-1 activity is regulated by S1PR1/3/c-Src-dependent EGFR and PDGFR transactivation, leading to an increased level of ICAM-1 [34]. The discrepancy could be due to different conditions of experiments and cell types.

PI3K/Akt phosphorylates and activates a range of downstream signaling pathways to regulate several important intracellular functions such as cell survival and proliferation [39]. PI3K/Akt is activated by both RTKs such as EGFR and PGFR and GPCRs [40]. Accumulating evidence showed that S1P can activate the PI3K/Akt pathway through RTKs [10, 34]. Further, our study dissected the role of PI3K/Akt in EGFR-mediated increased levels of COX-2 induced by S1P. Our previous data have revealed that S1P upregulates the expression of COX-2 via S1PR coupling to the G protein-mediated PI3K/Akt pathway in rat vascular smooth muscle cells [10]. In addition, S1P stimulated the PI3K/Akt/mTOR pathway to increase the protein level of COX-2 in human tracheal smooth muscle cells [41]. Aforementioned reports indicated that in various types of cells, PI3K/Akt contributes to COX-2 induction. Our data derived from the present study showed that, in HCFs, PI3K/Akt cascade participated in regulating S1P-induced COX-2 upregulation, since pretreatment with LY294002 (a PI3K inhibitor) or transfection with either p110 or Akt siRNA markedly attenuated S1P-mediated responses. We further revealed that S1P-stimulated Akt phosphorylation is required for COX-2 upregulation, which is attenuated by pretreatment with MMP2/9i, CMR-197, AG1478, or LY294002. These results suggested that in HCFs, PI3K/Akt cascade activated by S1P is crucial for COX-2 upregulation and PGE_2_ synthesis.

MAPK activation has a pivotal function in the expression of inflammatory genes triggered by a variety of injuries and inflammation [11, 36, 42]. An increasing body of evidence reveals that MAPKs stimulate diverse signaling pathways leading to upregulation of COX-2 expression [10, 43]. MAPK cascades have been disclosed to be an important effector for mediating S1P action in different types of cells [6, 10]. Our recent research has indicated that PKC-dependent activation of MAPKs (JNK1/2, p38 MAPK, and p42/p44 MAPK) contributes to the level of COX-2 in HCFs challenged with S1P [15]. PI3K/Akt is also a modulator of MAPKs which contributes to the induction of COX-2 in different types of cells triggered by various stimuli [10, 35]. In this study, we further clarified that in HCFs, COX-2 expression stimulated by S1P, MAPKs have an important modulating role, which is activated by upstream signaling molecules PI3K/Akt. LY294002, a selective inhibitor of PI3K, also markedly attenuated the phosphorylation levels of JNK1/2, p38 MAPK, and p44/p42 MAPK stimulated by S1P in HCFs. These results implied that PI3K/Akt-dependent MAPK activation plays a crucial role in increasing the level of COX-2 in HCFs exposed to S1P.

It has been well recognized that extracellular stimulus-triggered expression of several inflammatory genes is highly dependent upon AP-1 activation, one of the transcription factors [44]. The regulatory elements of *cox-2* genes located in the 5′-flanking regions contain several sequence elements that served as binding sites of various transcription factors such as AP-1/cyclic adenosine monophosphate- (cAMP-) response element (CRE, −59/−53) and NF-*κ*B (−223/−214), which are crucial for regulating *cox-2* gene transcription [45]. Therefore, activation of transcription factor AP-1 by external inflammatory stimuli might regulate the COX-2 transcription in numerous types of cells [12, 46]. These reports indicate that AP-1 contributes to the level of COX-2 expression related to the pathogenesis of inflammation. Our recent report in HCFs has indicated that MAPKs (p38 MAPK and JNK1/2, not ERK1/2)-dependent NF-*κ*B activity contributes to upregulation of COX-2 stimulated by S1P [15]. In the present study, our findings further showed that AP-1 subunit c-Jun participates in the S1P-induced upregulation of COX-2 and an increased generation of PGE_2_ since these responses were attenuated by pretreatment with tanshinone IIA (a selective AP-1 inhibitor) or transfection with c-Jun siRNA in HCFs. Moreover, we found that S1P boosted the ability of AP-1 to bind to the COX-2 promoter region, which was significantly blocked by AG1478, LY294002, tanshinone IIA, and the MAPK inhibitors including PD98059 (MEK1/2), SP600125 (JNK1/2), and SB202190 (p38 MAPK), suggesting that in HCFs, S1P-activated AP-1 is mediated through EGFR/PI3K/Akt-mediated MAPK (JNK1/2, ERK1/2, and p38 MAPK) activity-dependent mechanisms. Interestingly, MMP9 and HB-EGF are involved in the S1P-stimulated activation of AP-1 in HCFs, suggesting that EGFR transactivation is mediated through MMP9/HB-EGF in HCFs. This study also verified that the involvement of MAPKs in COX-2 upregulation induced by S1P is mediated through AP-1 activity. Our data also revealed that S1P-stimulated transcriptional activity of AP-1 was significantly blocked by abrogating S1PR1/3 and PTX-sensitive G_i_ protein or G_q_ activity, indicating that S1P-activated AP-1 activity is mediated through PTX-sensitive G_i_ or G_q_ protein-coupled S1PR1/3-dependent cascades. These findings are consistent with a recent study in human primary myometrial cells indicating that the increased level of COX-2 and cytokine release triggered by neuromedin B is mediated through AP-1 activation [47]. In addition, we constructed an AP-1 point-mutant of COX-2 promoter plasmid, and COX-2 promoter activity measured by the luciferase reporter assay was reduced while S1P stimulation as compared with that of wild type. Based on these findings, we revealed that in HCFs, MAPKs-dependent AP-1 activation plays a pivotal role in S1P-stimulated COX-2/PGE_2_ upregulation.

S1P is one metabolic product of complex sphingolipid and a bioactive sphingolipid metabolite. In general, S1P protects against apoptosis and stimulates cell proliferation, in contrast, sphingosine and ceramide promote apoptosis [48]. A growing body of literature points out a profibrotic effect of S1P signaling, although its role in cardiac fibrosis is still controversial. The postinfarction myofibroblast phenoconversion responsible for excessive ECM deposition is a phenomenon of quiescent cardiac fibroblasts activated following ischemic injury; it participates in the repair and remodeling of the ischemic heart injury. Evidence indicated that overexpression of sphingosine kinase 1 (SphK1) caused myocardial degeneration and cardiac fibrosis, which was mediated through an “inside-out” S1P/S1PR/Rho kinase signaling [49, 50]. S1P modulates cellular functions by two distinct mechanisms, either as an intracellular messenger or as a ligand of a family of GPCRs. There are some reports showing that S1P has apoptotic and growth-inhibitory effects related to the caspase-3 pathway in various types of cells dependent on cell density and different pathways being engaged [22, 23], although S1P generally elicits mitogenic and antiapoptotic effects. COX-2 could trigger neuronal loss induced by neurotoxicant trimethyltin and possibly related to the caspase-3 apoptotic pathway [51]. Carbon tetrachloride- (CCl_4_-) induced muscle injury is also caused by caspase-3 and COX-2 induction [52]. Moreover, advanced glycation end-products (AGEs) activate NF-*κ*B activation to upregulate the COX-2/PGE_2_ system, which causes apoptosis of pancreatic islet microvascular endothelial cells [53]. Consistently, our data derived from this study showed that S1P or PGE_2_ has antiproliferative potential in HCFs, which is rescued by blocking COX-2 activity (NS-398) or caspase-3 (z-DEVD). In contrast, other studies showed that selective COX-2 inhibitors can have proapoptotic and proliferative potential in various types of cells [54–56]. These inverse findings could be due to experimental conditions or cell-specific differences. The strengths of the study are that we used the gene downregulation to ensure the findings obtained from pharmacological inhibitors and the role of transcription factor AP-1 was proved using ChIP and promoter activity assays with the point mutation of AP-1. Moreover, this is the first time to address the comprehensive mechanisms underlying S1P-induced COX-2 overexpression and PGE_2_ production associated with apoptosis in HCFs. However, the limitations of the study should be a shortage of *in vivo* studies to expand the findings for further exploration and functional assay of MMP9 to be performed.

## 5. Conclusion

We concluded that the stimulatory effects of the S1P/S1PR system on *cox-2* gene expression are exerted through GPCR-mediated transactivation of EGFR in HCFs. S1PR1/3-coupled PTX-sensitive G_i_ protein or G_q_ protein sequentially activated MMP9, HB-EGF, EGFR, PI3K/Akt, MAPKs, and AP-1 signaling pathways and cooperatively implicated in the COX-2/PGE_2_ induction and apoptosis triggered by S1P. Based on our study and previous results, Figure 8 illustrates the molecular signaling mechanisms by which S1P induces gene overexpression of *cox-2* and PGE_2_ production in HCFs. These main findings of the increased level of COX-2 and PGE_2_ production induced by S1P are mediated through MMP9/HB-EGF-dependent EGFR transactivation, PI3K/Akt, MAPKs (i.e., JNK1/2, p42/p44 MAPK, and p38 MAPK), and AP-1 cascade, which leads to activating apoptotic factor caspase-3 in HCFs, implying that the cascade of S1P/S1PR might have a crucial function in cardiac inflammation. These results reveal that HCFs could play a key role as inflammatory cells which in heart diseases contribute to the inflammatory responses through the production of inflammatory mediators, besides their organized and preservative functions. Moreover, these results further clarify the potential role of S1P in cardiac inflammatory disease-related pathogenesis and provide advanced insights for creating effective strategies in cardiac disorders.

## Data Availability

The data used to support the findings of this study are available from the corresponding author upon request.

## Abbreviations

ChIP: Chromatin immunoprecipitation
EGFR: Epidermal growth factor receptor
GPCR: G protein-coupled receptors
HB-EGF: Heparin-binding epidermal growth factor
HCF: Human cardiac fibroblast
MMP: Matrix metalloprotease
PDK: Phosphoinositide-dependent kinase
PGE_2_: Prostaglandin E_2_
S1P: Sphingosine 1-phosphate
XTT: 2,3-Bis-(2-methoxy-4-nitro-5-sulfophenyl)-2H-tetrazolium-5-carboxanilide.
